# A Wearable Mobile Sensor Platform to Assist Fruit Grading

**DOI:** 10.3390/s130506109

**Published:** 2013-05-10

**Authors:** Rafael V. Aroca, Rafael B. Gomes, Rummennigue R. Dantas, Adonai G. Calbo, Luiz M. G. Gonçalves

**Affiliations:** 1 NatalNet Laboratory, Technology Center, Federal University of Rio Grande do Norte (UFRN), 59078-900 Natal, RN, Brazil; E-Mails: rafaelaroca@ieee.org (R.V.A.); rafaelufrn@gmail.com (R.B.G.); rudsondant@gmail.com (R.R.D.); 2 Embrapa Instrumentation, Brazilian Agricultural Research Corporation, Rua Quinze de Novembro, 1452, P.O. Box 741, 13560-970 São Carlos, SP, Brazil; E-Mail: adonai.calbo@embrapa.br

**Keywords:** ubiquitous computing, mobile sensing, glove-based platform, fruit grading, wearable computing

## Abstract

Wearable computing is a form of ubiquitous computing that offers flexible and useful tools for users. Specifically, glove-based systems have been used in the last 30 years in a variety of applications, but mostly focusing on sensing people's attributes, such as finger bending and heart rate. In contrast, we propose in this work a novel flexible and reconfigurable instrumentation platform in the form of a glove, which can be used to analyze and measure attributes of fruits by just pointing or touching them with the proposed glove. An architecture for such a platform is designed and its application for intuitive fruit grading is also presented, including experimental results for several fruits.

## 1. Introduction

Measurement systems and devices are present everywhere in the current world, but frequently based on computers and interfaces that need explicit user interactions. Several devices are not portable or user friendly, so they can be only used in structured laboratory environments by trained people. As we use hands for most of our daily tasks [1,2], one interesting solution is to integrate sensors in our hands to build a wearable mobile sensing platform in the form of a glove. The advantage of such platform is that the hands of the user and his attention become free for his task while the glove acts as an intuitive supporting device that provides and collects information about the task being done.

We propose a novel mobile sensing platform mounted on a glove that integrates several sensors, such as touch pressure, imaging, inertial measurements, localization and a Radio Frequency Identification (RFID) reader. As a platform, the system is suitable for several applications, but this work focuses on a fruit classification and grading system. One interesting usage of this system is help workers during manual harvesting.

### 1.1. Fruit Classification

It is known that agricultural products such as fruits and vegetables have, from the harvest moment until they are consumed, losses in quantity and quality of up to 25% in developed countries and losses of up to 50% in developing countries [3]. A more recent publication indicates that these losses can reach 72% in some Asian countries [4]. One of the reasons for such losses is the deterioration caused by physical damages [3], so firmness is essential for fruits to bear injuries during transport and commercialization [5]. Such damages can occur also during classification and other sorting processes in supply chains.

As said, horticultural products go through several sorting/handling processes: in the field, in the warehouse, in the distributor and in the market. Each classification involves mechanical manipulation of the product, which may cause additional damages and speed its deterioration [6]. Moreover, if the agricultural product is harvested at the ideal maturity, it will have longer shelf life and offer better quality [6–8]. On the other hand, if the product is harvested immature, it can be easily damaged during handling [9]. In that way, if a good classification is made at the harvest moment, the product can be stored in an identified box that will follow to the market, reducing the need of several reclassifications and manipulations.

In the fruit classification application, our glove uses data from several sensors while the hand of the user is approaching and touching a fruit. After the user touches the fruit, he is advised via tactile or audible feedback if that fruit should be harvested. The importance of such feedback is to help manual harvest workers to collect fruits at the best moment, trying to offer products with longer shelf life and better quality.

### 1.2. Contributions

The main contributions of this work are:

● A comprehensive state of the art review of glove-based systems and a brief review of fixed and portable fruit classification systems;
● A novel wearable mobile sensing platform;
● Usage of force/pressure sensors at fingertips to measure the hardness and pressure of objects, especially fruits;
● Hybrid palm-based optical sensing subsystem;
● Real time algorithms for interactive object segmentation and computation of its geometrical properties;
● Application of the proposed platform for intuitive and non-destructive fruit classification.

### 1.3. Organization

This paper is structured as follows. Section 2 describes the state of the art in glove-based systems and a brief overview of fruit classification systems, including some portable devices. Next, Section 3 describes the architecture of the proposed platform and the algorithms used with some practical and experimental considerations of its implementation and construction. Finally, a fruit classification study case application with experimental results is described in Section 4 and the conclusions are presented in Section 5.

## 2. Related Work

This section presents a review of articles and patents of glove-based devices in several applications. Some authors consider that Mann was one of the first researchers to present a wearable computing system [10], where computers would be an unobtrusive extension of our bodies providing us ubiquitous sensing abilities. Mann himself argues that his wearable computer was not the first one, and explains that there were shoe-based computers in the early seventies [11]. He says that in the future, “our clothing will significantly enhance our capabilities without requiring any conscious thought or effort” [11].

As one of the novelties of our work is the usage of the proposed glove for fruit classification, we also present a review of non-destructive techniques for fruit classification focused on techniques related to the ones used on our system.

### 2.1. Glove-Based Systems

As our interaction with objects in the physical world is mainly performed through our hands, many works in glove-based systems have been done in the last 30 years [2]. Dipietro *et al.* present a review [1] with several applications of glove-based systems in the areas of design and manufacturing, information visualization, robotics, arts, entertainment, sign language, medicine (including rehabilitation), health care and computer interface.

Gloves are especially used as input devices for wearable computers, which have a different paradigm of user interfaces. In such systems, the interaction with the computer is secondary because the user is mainly concerned with a task in the physical world [12]. Smart gloves or data gloves used as input devices are presented as alternatives to standard keyboards and mice both for desktop and wearable computers in several works [12–21]. These systems use sensors to sense discrete (yes/no) fingertip touches, and some use finger bending sensors and accelerometers to capture hands' and fingers' orientation.

There are several glove applications as assistive devices. For people with blindness, for example, a glove with ultrasound sensors can measure the distance to objects and emit tactile feedback [22]. Also for blind people, there is the possibility of identifying objects using a linear camera fixed to the tip of one finger [23]. Another glove-based system has special keys located at the fingers to allow text input in Braille [24]. Culver propose an hybrid system with an external camera and a data glove with accelerometer and finger bending sensors to recognize sign language [25]. For people unable to speak, it is possible to use gloves with speech synthesis to translate gestures into spoken sentences [26]. Gollner *et al.* describe a glove for deaf-blind people that uses several pressure sensors on the palm to understand a specific sign language and also to provide textual information to the user via tactile output through vibrator motors [27].

More recently, Jing *et al.* develop [28] the Magic Ring, a finger-worn device to remotely control appliances using finger gestures. In the same epoch, Nanayakkara *et al.* present the EyeRing [29], a finger-worn camera for visually impaired people with applications for recognizing currency notes, colors and text using optical character recognition (OCR). The camera worn in the finger sends live images to a mobile phone using Bluetooth, and the image recognition and processing tasks are done on the phone.

An increasing usage of smart gloves is in medicine and rehabilitation applications. Several systems were proposed and developed in order to measure hand and fingers posture [30–36] to help medical doctors diagnose certain patient's problems such as Parkinson's disease [37]. These systems are based on accelerometers, touch sensors, pressure sensors and finger bending sensors. In some of these systems, sophisticated sensors are used to measure bending, such as optical fiber sensors and Conductive Elastomer materials directly printed in the glove's fabric. Other systems add sensors to capture biological signals such as heart rate, skin temperature and galvanic skin response [38].

Related to medicine, there are also several health care applications. Angius and Raffo present a glove that continuously monitors a person's heart rate, and automatically calls a doctor if some risk situation emerges [39]. In the same way, another system calls a doctor if a glove detects an inadequate value of heart rate, oxygen in the blood, skin temperature or pressure of elderly people [40]. Another glove [41] has lights and a camera at fingertips to ease manual tasks related to health care and other delicate applications.

Several gloves have been built with an integrated Radio Frequency Identification (RFID) reader recently. RFID readers emit a radio signal to a low cost label (or transponder) that answers with an identification, frequently without the need of batteries, so objects can be easily and reliably identified by automation systems. Fishkin *et al.* propose a glove to study and analyze person-object interactions using a RFID reader fixed to the glove [42]. In a similar application, Hong *et al.* propose a RFID-based glove to recognize elderly people activities [43]. Another project also uses a RFID reader fixed to a glove and a probabilistic model to recognize daily tasks [44]. Im *et al.* propose a daily life activities recognition system using three accelerometers, a RFID reader, and several objects with RFID tags [45]. Min and Cho add gyroscopes and biological signal sensors to classify motions and user's actions in daily life activities [46]. Yin *et al.* present a system that analyzes human grasping behavior using electrical contacts at fingertips [47].

As a tool to improve and facilitate manual tasks, there are gloves with cameras attached at the back of the hand [48,49] to allow users to take pictures using finger gesture commands while keeping the hands free. One application, for example, allow policemen driving motorbikes [49] to rapidly take pictures when needed. Another usage of gloves as a productivity tool is the usage of a RFID reader fixed to the glove to help doing warehouse inventories [50].

Other glove systems are used for training and teaching, such as a glove that analyses the user's swing in Golf games [51] using pressure sensors. Pressure sensors are also used in a glove for music teaching, which uses tactile information, vibrating the user's fingers as they need to press piano's keyboards [52]. Similarly, Satomi and Perner-Wilson describe a glove with pressure sensors at fingertips to sense the force applied to the keys by piano students [53].

For security applications, a glove can detect if a vehicle driver is sleeping by measuring grip pressure and emit an alarm if needed [54]. Walters *et al.* present a glove to help firefighters sense external temperature, as it is hard for them to perceive the temperature given their thick gloves, obligating them to remove the gloves from time to time to sense the ambient temperature [55]. Ikuaki proposes a conjunct of glove and camera connected by a wireless link that can capture tactile information (temperature and object pressure) associated to a photo taken [56].

Table 1 shows a summary of the mentioned works divided by application categories. We note that several glove-based systems are not mentioned here, including several commercial products and open source/hobby projects. One interesting open source project worth mentioning is the KeyGlove [16], which aims to build a glove as a platform for several usages. At the moment, the KeyGlove can be used as an input device (mouse/keyboard).

From the presented review, it is clear that most glove-based systems are focused on studying people's behavior, as input devices or as a productivity tool. Furthermore, each of these systems is restricted to specific tasks and applications. In contrast with that, the wearable mobile sensing platform proposed in this work is focused on studying objects and their characteristics. Thus, although our focus is on its use in a fruit grading application, the proposed glove has several sensors that allow it to implement the applications of similar systems described in this review. For blind people, for example, our system can measure the distance to an object that a hand is pointing, and can also recognize objects using computer vision and RFID, or even hand gestures, among other examples.

### 2.2. Fruit Classification Systems

As detailed in Section 4, our aim is to embed several sensors in the proposed glove for non-destructive measurement of horticultural products' maturity. Even with the increasing usage of automated harvesting machines, one motivation for the usage of a glove to support harvesting is that manual hand-harvesting is still cheaper and widely used, however the quality of the collected products is subject to the workers judgement.

Harvesting fruits and vegetables at the right ripening moment is directly related to their resulting quality and shelf life [6,8]. Fruit quality can be evaluated using several techniques that measure internal variables such as firmness, sugar content, acid content and defects or external variables such as shape, size, defects, skin color and damages [6,8,59,60]. Diameter/depth ratios are also used as quality factors [60]. According to Kader, “Maturity at harvest is the most important factor that determines storage-life and final fruit quality” [7]. Kader also says that immature fruits, or fruits collected too soon or too late, offer inferior flavor and quality and are more subject to disorders. Moreover, Zhou *et al.* explain that injured fruits should be detected as soon as possible because they can get infected by microbes and spread the infection to a whole batch [61].

In general, fruits and vegetables can be classified using invasive or non-invasive techniques. Invasive techniques rely on inserting probes inside the product under analysis or extracting parts of it, while non-invasive techniques are only based on measurements taken externally. Another approach is called non-destructive evaluation, which might consist in the use of sensors that touch the fruit but without destroying it. We note that the penetrometer test, a destructive and invasive method, is still frequently used. The measurement is made using a device that has a probe that penetrates the fruit to read its firmness [6].

#### 2.2.1. Imaging Techniques

Appearance is considered one of the most common ways to measure quality of any material [62]. Gunasekaran argues that among several methods to evaluate food quality, computer vision is the most powerful [63].

Chalidabhongse *et al.* use a sequence of 2D images taken from various angles to build a 3D representation of mango fruit, implement 3D reconstruction for measuring geometrical properties such as area and volume with accuracy between 83% and 92% [64]. Charoenpong *et al.* use two cameras in different positions to obtain mango fruit volume with a root mean square (RMS) error of 2% and coefficient of determination of 0.99 when the data is fitted to real mango measures [65].

Mustafa *et al.* propose a system for measuring banana perimeter and other geometrical information [66]. As it is not possible to compute real geometrical information using only camera information, they place a dollar coin on the same image of the evaluated banana. As the diameter, area and perimeter of the coin are known in the real world, their computer vision system uses this information to compute the banana's parameters. They also use color to estimate ripeness. Lee *et al.* argue that color is often the best indicator of fruit quality and maturity, then present a color quantization technique for real-time color evaluation of fruit quality [67]. Their system performance reaches 92.5% accuracy for red fruits, and 82.8% for orange fruits.

#### 2.2.2. Mechanical Techniques: Firmness

Among several mechanical properties of fruits, firmness is considered one of the most important quality parameters [59,68]. Traditionally, firmness measurements are based on invasive systems, such as the penetrometer. One classical and well known technique for such test is the Magness Taylor, which measures maximum penetration force [59]. Looking for non-destructive firmness measurements, researchers proposed several alternative techniques to measure firmness. Several of them are mechanical, and some are based on spectroscopy and ultrasound.

One interesting alternative has been proposed by Calbo and Nery [5]. They present two devices for simple, accurate and non-destructive measurement of fruit firmness, based on cell's turgor pressure, which is known as the pressure difference between cell interior and the barometric pressure [69]. Their systems consist of devices with a base to place the fruit, and a moving applanating plate that rests on the top of the fruit. After placing the applanating plate on the top of a fruit, the user waits 1 or 2 minutes to read flattened area at presumed constant cell pressure. Alternatively, these systems allow immediate flattened area readings at presumed constant cell volume [70] to calculate firmness as a ratio between the applied external force and the fruit flattened area.

Although simple and practical, Calbo *et al.* explain that this method and variations of it are still not used in commercial instruments to measure turgor [69]. As a possible solution, they recently proposed a miniaturizable sensor system for measuring cell turgor, an important fruit firmness component that is used in this work as explained in Section 4. Their system can be used to build a hand-held device that can be manually pressed against fruits on the market to instantly obtain fruit firmness [69]. According to these authors, mature fruits should present pressures ranging from 0.2 to 1 kgf/cm^2^ and mature green fruits present pressures from 1.5 to 4 kgf/cm^2^. In all cases, pressure values close to zero means that the fruit is inadequate to be consumed.

The device proposed by Calbo *et al.* [69] is suitable even for organs with irregular surfaces, such as cucumber and oranges, however care should be taken during the usage of devices based on this principle regarding the compression applied by the user. The fruit under evaluation must be pressed in a way that it must be completely in contact with the sensing area of the sensor. This can be easily achieved using force or pressure sensors with small sensing areas.

Thanks to the development of embedded technology, real time image processing systems in the form of compact and mobile devices are possible today [71]. Some works relate the usage of mobile computer vision systems to measure geometrical attributes of 2D objects with 2 mm precision [71].

Wong *et al.* propose a mobile system to grade fruits using user's mobile phones. In their system, the user can take a photo of a fruit and send it using MMS (Multimedia Messaging System) to a server, which will process the photo taken and send a Short Message (SMS) to the mobile phone with the fruit grading. A coin of known size must be in the same photo so that the grading algorithm can compute real geometrical properties of the fruit based on the known size of the coin. Due to transmission timing, the response is just received some minutes after the photo has been taken [72].

## 3. Wearable Sensing Platform Architecture and Implementation

**Figure 1.**
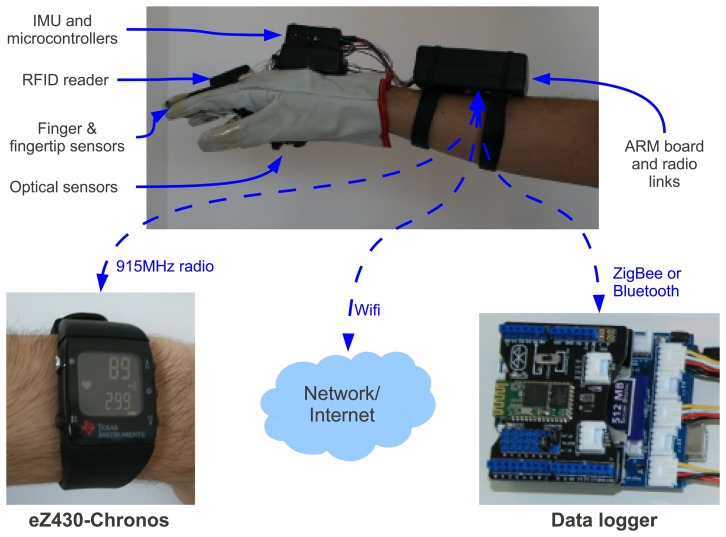
Overview of the system and its communication with external devices.

Figure 1 shows an overview of the proposed platform and its communication with external devices. The glove operation is standalone, without the need of any of these external hardwares, but if any is available, the glove can use them as auxiliary devices.

As seen in Figure 1, external devices communicate with the glove via the ARM board inside the box in forearm of the user. The ARM board is based on a Gumstix Overo FireSTORM module, which runs the main algorithms of the system on a 800 MHz ARM Cortex-A8 processor with 512 MB of RAM and 8 GB SD Card. This box also contains WiFi, Bluetooth and 8 AAA standard batteries. The processor runs Linux with the OpenCV computer vision library.

**Figure 2.**
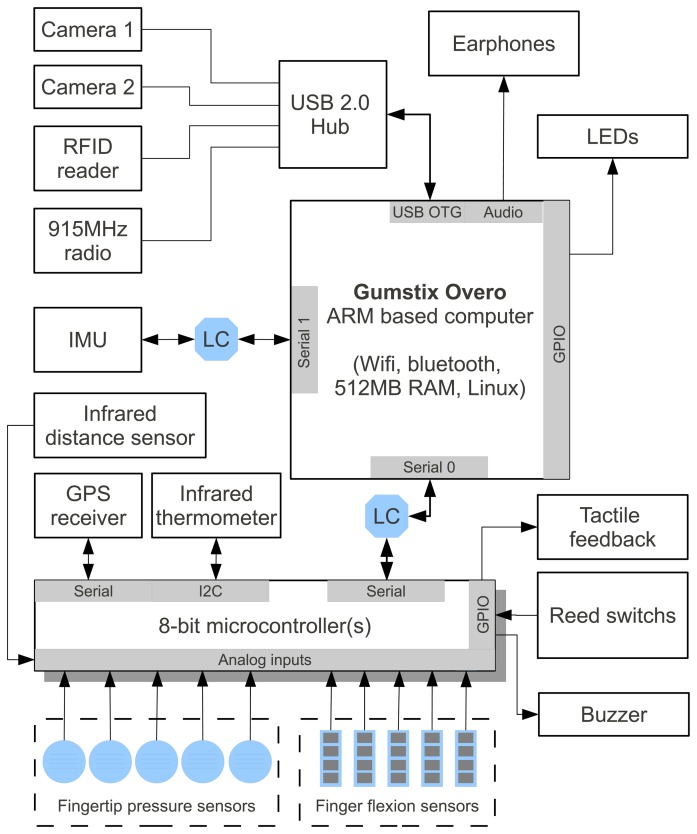
Wearable mobile sensing platform hardware block diagram.

Figure 2 depicts the hardware's block diagram. As said, the central unit of the system is the ARM processor, which is shown as Gumstix Overo in the block diagram. It has an Universal Serial Bus (USB) On-The-Go (OTG) port connected to a USB 2.0 HUB, which is installed in the box located at the top of the glove (see Figure 1). This HUB connects 2 USB cameras mounted in the palm of the hand, a RFID reader, also shown in Figure 1, and a 915 MHz radio to exchange information with a programmable wrist watch.

Figure 1 also shows the ez430-Crhonos programmable wrist watch from Texas Instruments, which can be used to easily display numeric information measured by the glove's sensors to the user. Another interesting feature is that the glove can connect to data loggers with wireless communication, such as Bluetooth, and use the information collected by the data logger during several days to aid its computation about a given object and its environment.

As the ARM processor has logic levels of 1.8 V and the microcontrollers and Inertial Measurement Unit (IMU) have logic levels of 5 V and 3.3 V, logic level converters are needed to interconnect them using asynchronous serial ports. In the block diagram, the level converters are shown as “LC”. All the sensors fixed to glove are connected to 8-bit microcontrollers (AtMega 328), so the only wirings from the box on the glove to the box on the forearm are serial lines. Next, a description of each module, sensor and auxiliary device is presented.

### 3.1. Finger Sensors

There are two types of sensors mounted on the fingers: finger bending sensors to measure finger flexion angle and pressure sensors mounted on each fingertip.

Finger bending sensors present a varying electrical resistance according to their bending. Each of them is connected to a voltage divider circuit (with a 10 *K*Ω resistor) and the divider output is connected to a LM358 operational amplifier to make the reading more robust and reliable. The operational amplifier output is then connected to the analog input of the AtMega 328 microcontroller. In order to calibrate the sensors, a user must wear the glove and completely close the hand while the system reads analog to digital converter (ADC) values for each finger several times. Next, the user completely opens the hand and the ADCs related to these sensors are read again. Then a linear function maps the angles between fingers completely closed or completely opened.

For the fingertip pressure sensors, the same electronics setup with voltage divider and operational amplifiers is used; however, the calibration procedure is different. We evaluated two types of sensors for fingertip pressure measurement: a probe connected to a pressure transducer by a flexible tube and Force Sensitive Resistors (FSRs).

**Figure 3.**
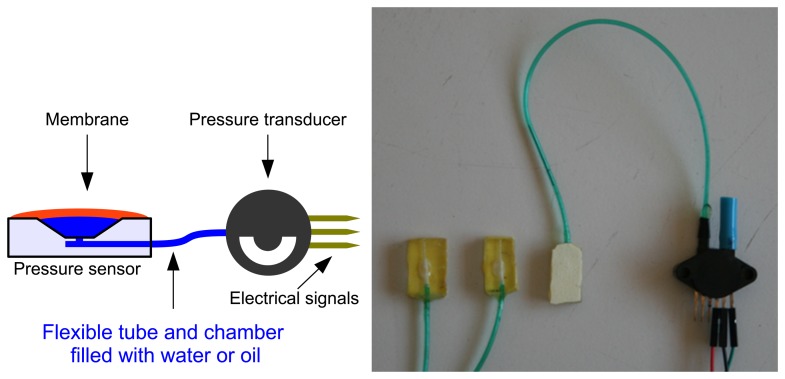
Possible miniaturizable setup for the usage of a pressure transducer to measure fingertip pressure while grasping.

Figure 3 shows the first possible setup that we have evaluated. In this system, a probe consists of a small piece of plastic material that is drilled to have a chamber and an output connected by a flexible tube to a pressure transducer. A membrane (yellow on the photo and red on the scheme) is glued on the top of the plastic piece to cover the chamber. The chamber and the flexible tube are completely filled with water or oil.

For the tests, a MPX5700 pressure transducer from Freescale was used. Given a 5 V DC power input, Equation (1) shows the transfer function provided by the manufacturer, where X is the sensor output tension in Volts, A is the offset, typically of 0.2 Volts, and 0.101972 is a constant to convert pressure from kPa (Kilo-Pascals) to kgf/cm^2^ (kilogram-force per centimeter squared).

(1) $$P_{0}(\text{kgf}/cm^{2})=\left(\frac{X-A}{0.064290}\right)0.101972$$

**Figure 4.**
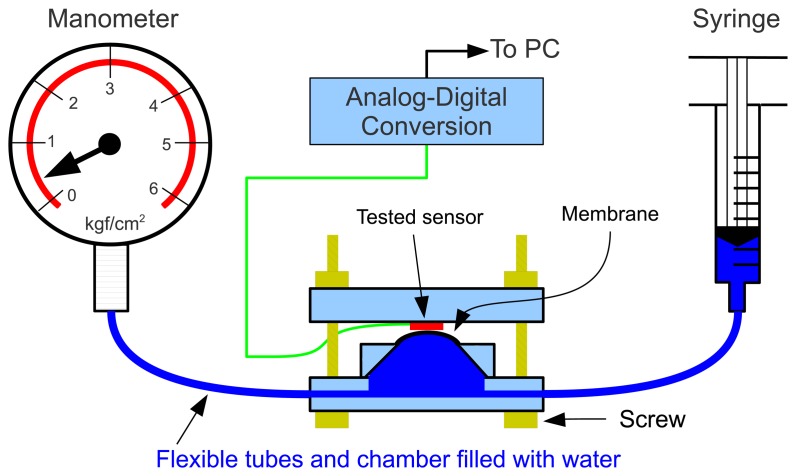
Calibration system for pressure sensors. Based on the leaf Wiltmeter developed by Calbo and Pessoa [73].

As there is a probe with a chamber and a flexible tube connected to the pressure transducer, a calibration of the entire system can be done for evaluation. In order to do this calibration, we built a calibration system based on the Wiltmeter [73] base gauge. In this system, the sensor to be calibrated (red rectangle in Figure 4) is inserted between two plates and gently pressed against these plates with screws. In the bottom plate, there is a chamber and a membrane contacting the sensor. For a reliable calibration, it is important that the sensing area of the sensor under calibration is fully covered by the membrane. After the sensor is tied, one must manually press the syringe and keep it pressed in a steady position for some seconds. During this time, the pressure is read in the manometer and the voltage from the sensor under evaluation must be also read.

This procedure was repeated for several values. In our case, we pressed the syringe to obtain pressure values from 0 to 6 kgf/cm^2^ in intervals of 0.5 kgf/cm^2^. The analog voltage reading of the sensors were taken automatically with a digital multimeter with RS-232 interface connected to a computer, so we were able to acquire 30 values for each pressure imposed with the syringe, and take the average of these 30 values to use later.

After performing the measurements, we found a linear relation of the pressure and our probe response. From Equation (2) the linear regression determination coefficient was *r*^2^ = 0.99. This equation allows easy mapping of the pressure measured by the probe to be used at fingertips.

(2) $$P\;(\text{kgf}/cm^{2})=0.83P_{0}-0.02$$

Although reliable and precise, there are some issues related to the use of the pressure transducer, such as the need of removing all air of the chamber and tube, which may be laborious due to the size of the parts and the transducer size. The current implementation of the glove used a simpler sensor for pressure measurements: the Force Sensitive Resistor (FSR). As the pressure definition is force applied to a certain area (F/A), if the sensing area is known, we can use a force sensor to obtain pressure.

**Figure 5.**
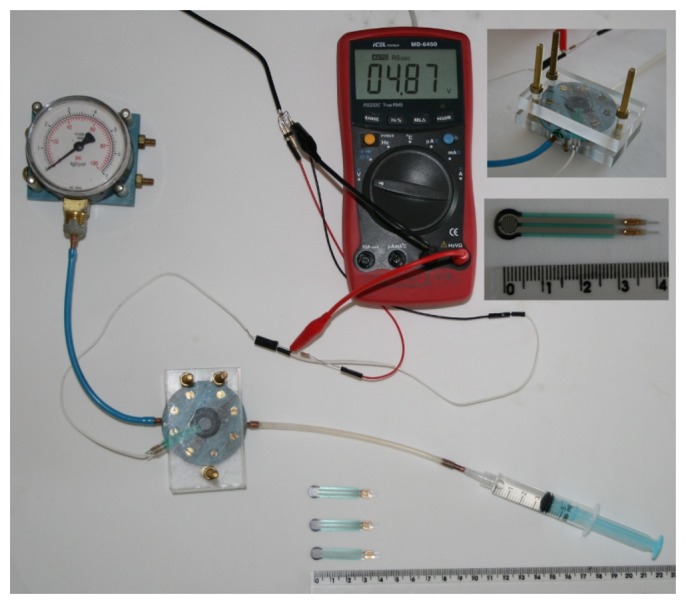
Calibration system in use with a FSR sensor.

Although less precise than the direct pressure transducer setup, the FSRs are easier to work with and highly thin/compact. They respond to force applied to their sensitive area by varying their electrical resistance according to an inverse power law. In order to calibrate these FSR sensors, we used the same calibration system already described and shown in Figure 4. Figure 5 shows a photo of a FSR sensor being calibrated, and a closer view of the FSR sensor (on the right).

**Figure 6.**
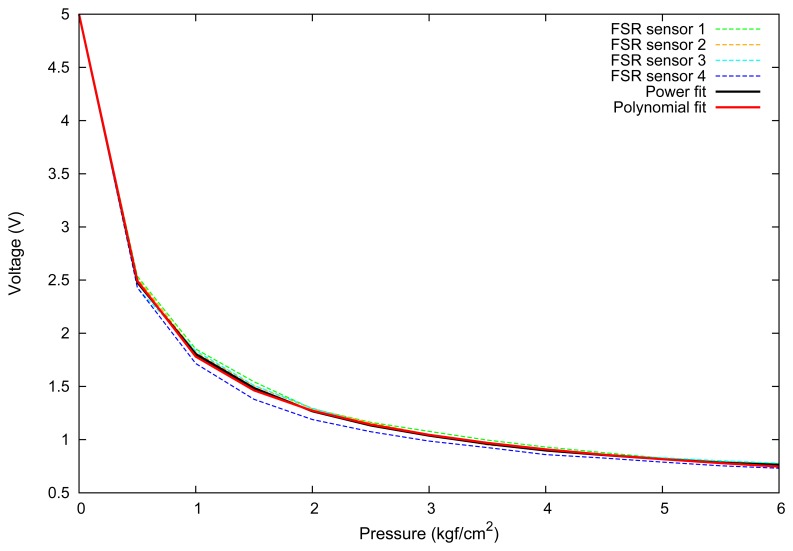
Calibration response of 4 FSR sensors and results for polynomial and power fit.

After acquiring all the values, we look for a mathematical expression that properly fit these values. For this purpose, we enter the acquired values in a power regression algorithm and in a polynomial regression algorithm and obtain the data fitting shown in Figure 6. This figure shows the calibration results for 4 different FSR sensors, and a power (black line) and polynomial fit (red line) for the average of all sensors calibration. The graph clearly shows that the power curve, as the theoretical response of this type of sensor, yielded, as expected, the best response.

Equation (3) shows the result of the second degree polynomial fit for the FSR sensor, which had a determination coefficient *r*^2^ = 0.95 and is shown in the plot of Figure 6.

(3) $$P(\text{kgf}/cm^{2})=0.0802x^{2}-0.77x+2.606$$

A better fit was obtained with a power fit, which had a determination coefficient of *r*^2^ = 0.99. The resulting transfer function that maps raw sensor values into pressure is described by Equation (4), where *x* is the tension in Volts from the sensor. This relation can be used for applications in medicine, rehabilitation and plant science. Moreover, given the pressure/force relation *P* = *F/A*, both quantities can be easily obtained.

(4) $$P(\text{kgf}/cm^{2})=3.29x^{-2.061}$$

Although inexpensive, FSR manufacturers highlight that such sensors are sensible for actuation forces as low as 1 gram, offering a wide pressure operating range (from less than 0.1 kgf/cm^2^ to more than 10 kgf/cm^2^). They also say that these sensors have good repeatability and high resolution, making them ideal for wearable applications.

### 3.2. Palm-Based Optical System

**Figure 7.**
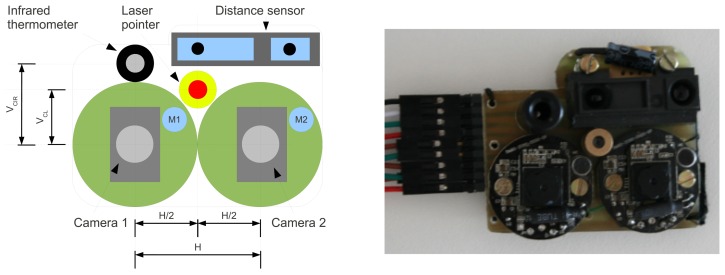
Optical sensors board scheme, and board built.

The objective of the optical board fixed in the palm of the hand is to acquire optical information about a given object to which the user points his palm. The board is composed of an infrared distance sensor (based on LED emitter/receiver with *λ* = 850 *nm* ± 70 *nm*), a long wavelength infrared (LWIR) thermometer (from *λ* = 8 *μm* to 15 *μm*), a laser pointer that can be turned on and off by software commands and a pair of VGA cameras. Figure 7 depicts each sensor mounted on this board and shows a photo of the board actually built. M1 and M2 are cameras' microphones that can be used to capture audio.

#### 3.2.1. Distance Sensor

The distance measurement is the first step of the computer vision mechanism of the platform. In order to save power, with the exception of the distance sensor, all other parts of this board are kept off by default. The distance sensor (SHARP GP2D120XJ00F) continuously monitors the distance from the optical sensors to objects in front of it. When an object is detected in the range from 5 cm to 25 cm, the microcontroller turns on the power of the optical sensors and starts computer vision analysis. Unfortunately, the output voltage of this sensor is not a linear function of the measured distance, so we collected thirty distance sample pairs (distance/voltage) and found the best fit using a power regression shown in Equation (5). In the equation, x is the raw ADC value, and D is the distance measured by the sensor.

(5) $$D(cm)=2736.24x^{-0.9909}$$

#### 3.2.2. Laser Pointer

The laser pointer module positioned between the 2 cameras can be seen in Figure 7. This device emits a focused red light beam with *λ* = 650 *nm* that appears as a very bright red filled circle in the object that the glove is pointing at. Its main usage is for interactive segmentation of objects. When the user points his hand to a certain object, the red dot projected on the object helps the computer vision software to segment this object from the rest of the scene faster and more easily.

**Figure 8.**
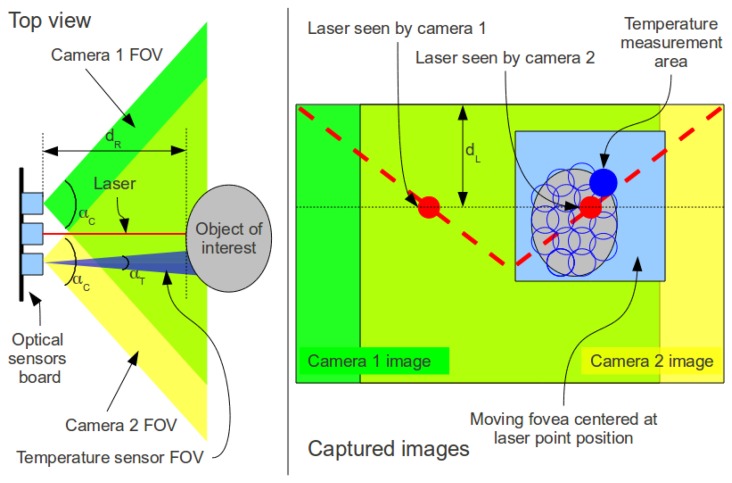
Optical imaging layout. Left image shows a top view diagram of the system and right image shows a captured image example.

Using a single camera does not make possible the computation of geometrical information of the real world. With the aid of the laser, it is known that the bright red dot projected on the object comes from a line parallel to the optical axis of the camera, which makes possible the computation of precise object distance, and based on that, other geometrical properties of the object. Basically the distance *d_L_* of Figure 8 is proportional to the object's distance. More details about distance measurement with cameras and laser pointers can be found in the work of Portugal-Zambrano and Mena-Chalco [74].

The small distance (baseline) from the laser to the cameras (H = 2.5 cm) allows only a small range to be measured with the laser (from 3 cm to 7.3 cm). In this range, the system uses distance computed using the laser pointer to obtain a more accurate distance measurement, and consequently more precise area, perimeter and other geometrical properties. One of the reasons for such approach is that the IR distance sensor is less precise than the laser based method.

#### 3.2.3. Dual Camera Head

The dual camera setup can operate in two modes: standard stereo or a composite of visible Red, Green, Blue (RGB) and Near Infrared (NIR). In the stereo mode, the cameras operate similarly to the human eyes. Their different points of view of the same scene cause a disparity that can be used to obtain depth information of the image to reconstruct the 3D scene. Detailed information about stereo vision can be found in most computer vision textbooks.

One of the major problems of a stereo vision system is that it can be considerably slow, and consequently unsuitable for the glove application. This happens because an algorithm must scan blocks of the image of the left camera and compare with blocks of image of the right camera in order to find correspondences between the images of the two cameras. Our solution for this sensing platform relies on the usage of the moving fovea approach proposed by Beserra *et al.* [75], which focuses the computation of the stereo disparity map only in a window that contains the object of interest, but not entire images, thus decreasing the processing time. To improve the performance even more, we use the laser point position as the center position of the moving fovea in both images.

The second operating mode consists in merging information from visible and invisible light spectrum. To do that, the standard lenses of one of the cameras must be removed and replaced by lenses with a filter that rejects visible light (from *λ* = 380 *nm* to *λ* = 750 *nm*) and allows near infrared light to enter the sensor (starting at *λ* = 750 *nm)*. The usage of mixed NIR and visible images is useful for several applications. Salamati, for example, explores mixing NIR and visible images to obtain information about material composition of objects in a scene [76]. Other authors use a combination of NIR and visible images to build more robust and accurate segmentation algorithms [77].

To integrate NIR and visible spectra, most systems use optical setups that split incoming light rays into two cameras with mirrors and lenses, but this setup is also not possible on our system due to size constraints. Our solution makes the assumption that the object of interest is planar, so the distance to all points in this object is the same. As the distance to the object is known from the distance sensor, we use the stereo vision equation to obtain the relation of the visible image pixels coordinates with the infrared image pixel coordinates. The cameras are horizontally aligned to take advantage of epipolar geometry [78], so all lines on one image correspond to lines on the other image.

Equation (6) shows the standard stereo vision equation, which is typically used to obtain depth (Z in the equation) by using known camera parameters, which are the focal length (f) and the distance between the cameras, also called baseline (H). As f and H are fixed, the variables that determine Z are *x_L_* and *x_R_*. The same point of the real image appears at different positions on the left (*x_L_*) and right images (*x_R_*). This difference is called disparity. To merge the NIR and visible images into a unique 4 channel image, we use Equation (6) as shown in Equation (7). The four channels are R (red), G (green), B (blue) and I. R, G and B come directly from the color image captured by one of the cameras, and I comes from Equation (7) computed with data from the modified camera to reject the visible spectrum.

(6) $$Z=\frac{fH}{x_{L}-x_{R}}$$

(7) $$x_{L}=x_{R}+\frac{fH}{Z}$$

Each pixel of the resulting 4-channel image *MI* is given by Equation (8). In this equation, the left camera captures visible images (*V IS*) and the right camera captures infrared images (*NIR*). The left image is taken as the reference, so for all x, *x* = *x_L_*. To improve the result of the 4-channel image, a calibration can be done.

(8) $$MI{\;(x,y)}_{\left(R,G,B,I\right)}=\text{V I S}{\;(x,y)}_{\left(R,G,B,0\right)}+\text{N I R}{\;(x-\frac{fH}{Z},y)}_{\left(0,0,0,I\right)}$$

The image acquisition process done by the cameras suffers from several factors that degrade the captured image. Apart from quantization and electrical noise, the optical distortion due to the lenses causes differences in the pixel sizes of the same image, especially when we compare pixel sizes from the image's center to its peripherals. This problem is even worse in our situation because we want to extract reliable geometric information from the object of interest. To alleviate this problem, we use Zhang's [79] camera calibration to reduce distortion.

Each camera operate with 640 × 480 (VGA) resolution and has a field of view (FOV) of 48 degrees. With this FOV, these cameras can capture objects with diameters of 28 cm at a distance of 31 cm and with diameter of 6 cm at a distance of 8 cm.

Figure 8 shows the optical layout of the sensors with their fields of view highlighted in blue, green and yellow. The left diagram shows a top view of the cameras capturing an object image, and the right diagram shows an example of the resulting images. Note that most of the scene intersects in both cameras. The figure also shows the remote temperature measurement accomplished by the infrared temperature sensor that measures the temperature of a given area proportional to its distance from an object.

#### 3.2.4. Object Segmentation and Seed Tracking

One paramount step that affects the quality and reliability of all remaining parts of the system is the object segmentation, which is a hard task given the non-structured and unknown environment that gloves might operate. As already said, we use the laser point to allow the user to interactively point which object to segment [80,81]; however, this approach might not be enough to keep the object correctly segmented while the user is moving his hand towards the object. To make the segmentation more reliable, we transform each detected laser point in a seed and keep control of each of these seeds until all desired information is computed from the image.

When the user moves his hand, the optical flow based on the Lucas–Kanade method [82] tracks the laser seeds (simply, all the places where the laser was pointed to for a certain time) to keep the object segmented according to the seeds deposited by the laser pointer. A time to live mechanism prevents spurious seeds to deteriorate the segmentation result. For a detailed discussion of the object segmentation with seed tracking, please refer to the work of Beserra *et al.* [81].

Based on the seed set, a fast Fuzzy [83] segmentation algorithm is executed to extract the object of interested from the rest of the scene. In the application example described in Section 4, examples of figures of the resulting segmentation are shown. The resulting segmentation has a large number of applications, such as object classification, recognition and counting.

### 3.3. Other Sensors and Actuators

As shown in Figure 2, the architecture also has several other multi-purpose accessories, briefly described here.

● GPS: A Global Positioning System (GPS) receiver that is powered off by default. It can be powered on by software when some glove application needs to obtain geographical location. An example application is to build thematic geographical maps of fruit quality and productivity.
● IMU: an Inertial Measurement Unit (IMU) is mounted on the top of the hand in order to obtain accurate and reliable Euler angles (roll, pitch, yaw) about the orientation of the hand. One application of the IMU is to stabilize and improve pictures taken by the cameras.
● As actuators to notify and give feedback to the user, there is a vibration motor that can be controlled to vibrate the glove with different timings, and a piezoelectric buzzer to emit sounds of different frequencies. The system can be adjusted to activate these actuators according to several values measured by the glove.
● An optional RFID reader fixed to the ring finger allows objects with RFID tags to be easily identified by the glove software by simply approaching the hand to about 5–15 centimeters from the object. The RFID reader can be seen in Figure 1. An example usage is to integrate the fruit grading system with automatic product history and tracking systems.
● A programmable wrist watch such as the ez430 Chronos can also be used to show information about the glove's sensors to the user. A possible use is to allow users to view quantitative information about the sensors.
● An infrared temperature sensor that remotely measures temperature is also present in the palm of the hand as shown in prior figures. It can be used during harvest to measure both ambient temperature and the objects' temperatures, such as fruit temperature, to aid posterior studies of fruit conditions and quality.

**Figure 9.**
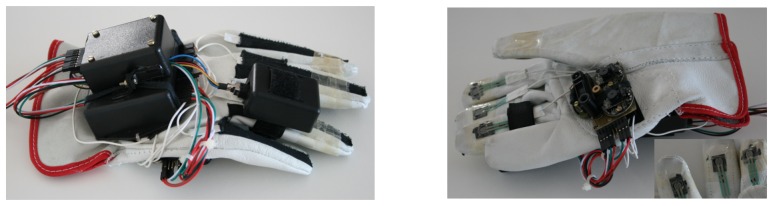
Top and bottom view of the glove. On the left are finger bending sensors, IMU and USB HUB. On the right there are the optical sensors and finger pressure sensors.

Figure 9 shows the top and bottom of the constructed glove prototype. We remind that the system is a fully functional prototype that was built with standard and simple tools. It can have its size considerably reduced using surface mounting devices (SMD) technology and custom made glove and sensors.

As a platform, we note that subsets of this system can be easily built. For example, one of the drawbacks of the computer vision system is that it consists of an intensive processing application while the cameras also have considerable power consumption (approximately 300 mA of current), leading to a short battery life. In our measurements, the system's battery life is of about 40 minutes when continuously performing computer vision tasks. We think this is acceptable as our platform is novel and a proof of concept, which may be considerably optimized with newer batteries, low power processor technologies and low power cameras such as the ones used in mobile phones.

As a useful alternative for several applications, the platform can be used without the computer vision sub-system and the ARM processor. In that case, only the 8-bit microcontroller is powered, and although the features are limited, several tasks can be done. We have made experiments with a possible setup without computer vision in which the system can measure pressure, temperature and finger bending. Such setup allows the system to function continuously for 108 h (about 13 work days of 8 h) with only two standard rechargeable 2,700 mAh batteries, or 48 h with two 1,200 mAh batteries.

## 4. Results for a Fruit Classification Application

The great number of applications and possibilities of glove-based systems were already discussed in Section 2. In this section we describe details and experimental results of a novel glove-based system for fruit classification.

### 4.1. Overview

The system we have implemented allows evaluation of fruit quality by simply pointing the glove to a fruit and then touching this fruit. It uses distance information and one of the cameras to compute fruit's area and then approximates its volume by a spherical model. Finally, when the user touches the fruit, the volume is also computed based on the finger bending sensors, its turgor pressure is measured and an overall quality parameter is computed according to weights for each of these parameters that can be provided by the user. If the result is below a certain customizable threshold, the tactile feedback system warns the user by vibrating the glove during a specified time.

### 4.2. Pressure Measurement

To perform the turgor pressure tests, fruits in different stages of development were purchased at a local Brazilian market. The purchased fruits are climacteric, which means that they are able to ripen after being picked. The fruits are: Tomato, Pear, Banana, Papaya, Guava and Mango.

**Figure 10.**
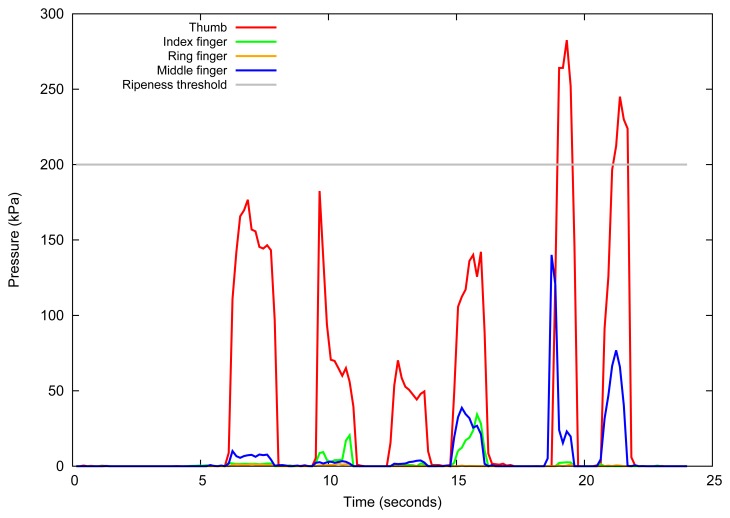
FSR sensor response during grasping of 6 tomatoes. Each peak represents the pressure of a grabbed tomato that is ripe, ripe, spoiled, ripe, unripe and unripe, respectively.

Figure 10 shows a plot of the turgor pressure of six tomatoes captured with the fingertip sensors during a period of time that comprehends several grasp operations in each tomato using the glove shown in Figure 9. The plot shows 4 lines, one for each finger with a FSR sensor, and each peak in the graph represents a tomato being grasped for some seconds and then released. It is important to note that the user does not have to apply a specific force to the tomato. One must only care to grab the tomato with fingertips in a way that the fruit will be completely touching the pressure sensor, and that the force is not too high to damage the fruit or too low such that the sensor will not sense the pressure. The gray line shows a threshold line of 200 kPa to classify the tomatoes as ripe or unripe. We remark that the threshold is specific for each fruit variety.

As the technique used is new, there are still no standard tables of values for such fruits, which is in the scope of a future work. Again, the threshold is not the same for different fruits and even for different varieties of the same fruit, however they fit in a range of pressures. In that case, unripe fruits present turgor pressures from 150 kPa to 400 kPa, and ripe fruits present turgor pressures from 20 kPa to 100 kPa [69]. Pressure values close to zero means that the fruit is inadequate to be consumed.

Figure 10 clearly shows that the measurement of some fingers must be ignored because their pressure is considerably lower than the ones acquired by other sensors, probably because these fingers did not cooperate well to the grasping. Furthermore, it also shows the pressure difference of ripe tomatoes, green tomatoes and spoiled tomatoes that are inadequate for consumption. In the figure, the first and second peaks correspond to ripe tomatoes, the third to a spoiled tomato, the fourth to a ripe tomato and fifth and sixth to ripening tomatoes. These results are consistent with the measurements made by Calbo and Nery using other devices [5]. Furthermore, according to Nascimento Nunes, this pressure measurement alone already offers an important quality index related with eating quality and longer post-harvest life of tomatoes [84].

**Figure 11.**
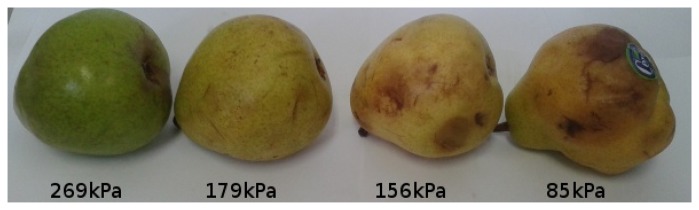
Turgor pressure in Kilo-Pascal (kPa) measured with the glove for peach. The fruit on the left is more unripe and the one on the right is more ripe.

**Figure 12.**
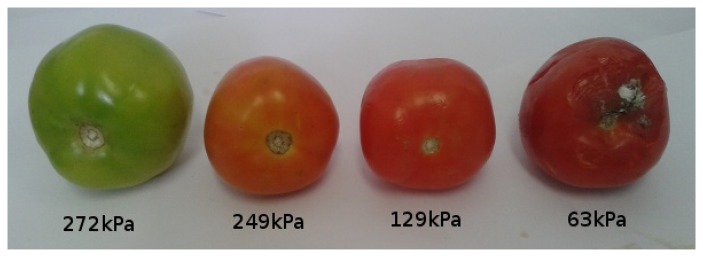
Turgor pressure in Kilo-Pascal (kPa) measured with the glove for tomato. The fruit on the left is more unripe and the one on the right is more ripe.

**Figure 13.**
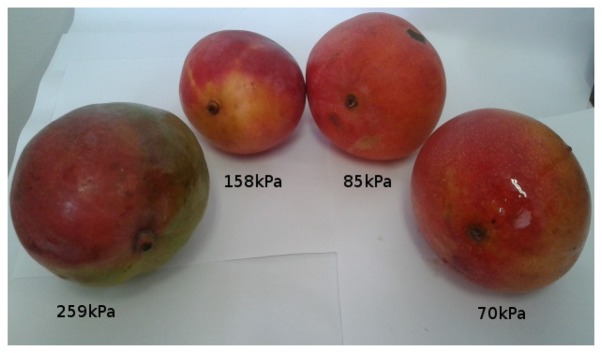
Turgor pressure in Kilo-Pascal (kPa) measured with the glove for mango. The fruit on the left is more unripe and the one on the right is more ripe.

**Figure 14.**
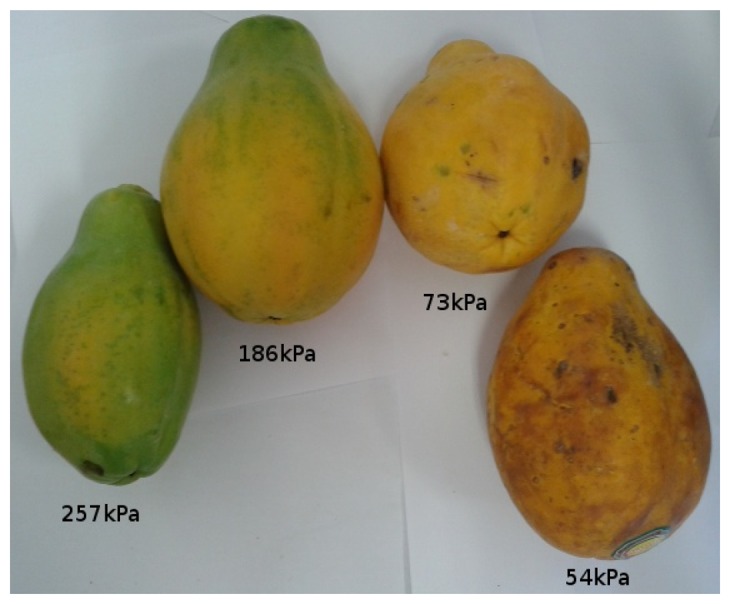
Turgor pressure in Kilo-Pascal (kPa) measured with the glove for papaya. The fruit on the left is more unripe and the one on the right is more ripe.

**Figure 15.**
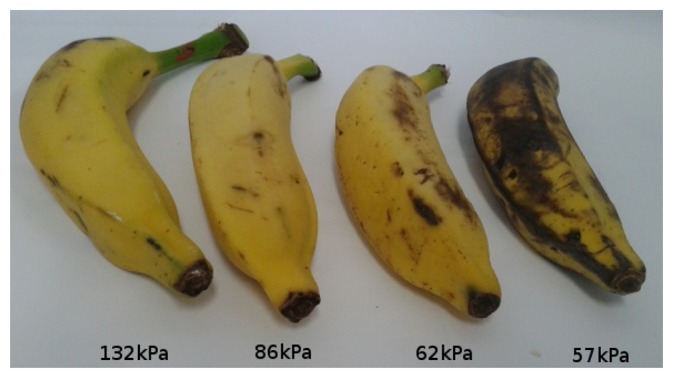
Turgor pressure in Kilo-Pascal (kPa) measured with the glove for banana. The fruit on the left is more unripe and the one on the right is more ripe.

**Figure 16.**
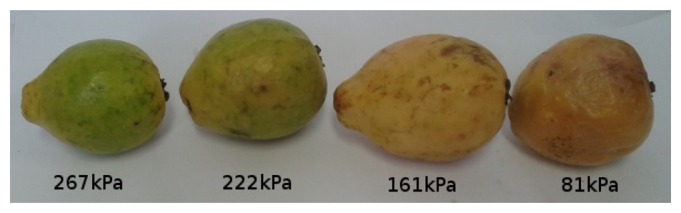
Turgor pressure in Kilo-Pascal (kPa) measured with the glove for guava. The fruit on the left is more unripe and the one on the right is more ripe.

Figures 11, 12, 13, 14, 15 and 16 show some of the fruits evaluated using the glove. Each figure shows four fruits: from the left to the right, the first is the most unripe, and the one on the right is the most ripe (considering the sample fruits). The figure also shows the respective turgor pressure measured for each fruit, which is the average of 30 measurements.

Table 2 shows detailed information about the measurements done, including the average of 30 measurements, maximum and minimum values and standard deviation. Note that the standard deviations are less than 10% of the measured value in all cases, showing reliability in the measurements done by the presented system.

### 4.3. Finger Bending Sensor

We also propose the usage of the finger bending sensor described in Section 3 to measure the diameter of a spherical object. For that purpose, an adjustment spline curve is passed through several calibration points and then several measurements are done for other spheres of different diameters in order to evaluate the overall performance of the system.

The values acquired for calibration are shown in Table 3, which shows the raw finger bending sensor reading, which can vary from 0 to 1,024, and the diameter of reference spheres used for sensor calibration.

**Figure 17.**
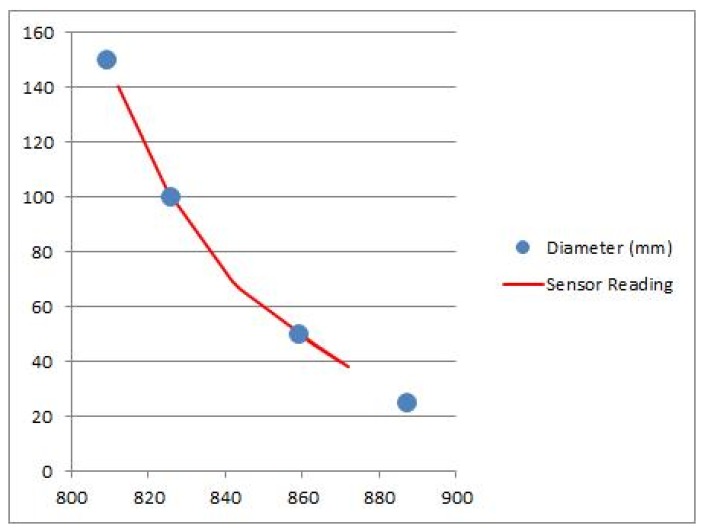
Spline curve adjustment to fit values from the finger bending sensors to spherical shapes.

Figure 17 shows the results of a spline curve fit to adjust the values from the finger bending sensors to the spherical shapes. Table 4 shows a list of several experimental measurements obtained with the glove, compared with the real value of the spherical objects (the Ground Truth). Note that the errors are as low as 3%.

Thus, we have shown the possibility of using the finger bending sensors to measure finger angles, and based on calibration data, use these angles to estimate the diameter of ideal spheres, which can be used to obtain sphere's radius, volume and other geometrical data. Such results could be used to estimate volume of spherical fruits.

### 4.4. Optical Measurements

As mentioned in Section 2, volume measurement is an important metric to evaluate fruit quality. In order to measure fruit's volume we use the Fast Fuzzy segmentation algorithm to extract the fruit's image from the rest of the scene. The fruit is selected by pointing the laser at it, and then the segmentation system searches for the brightest red point in the image and segments that region. After the segmentation, the system counts how many pixels were segmented in the image. The number of segmented pixels is the area of the object. To evaluate the real metric area, we make the assumption that the object is spherical and it is projected on the camera as a circle, thus Equation (9) can be used to compute the area of the circle, which can be manipulated to give its radius as shown in Equation (10).

**Figure 18.**
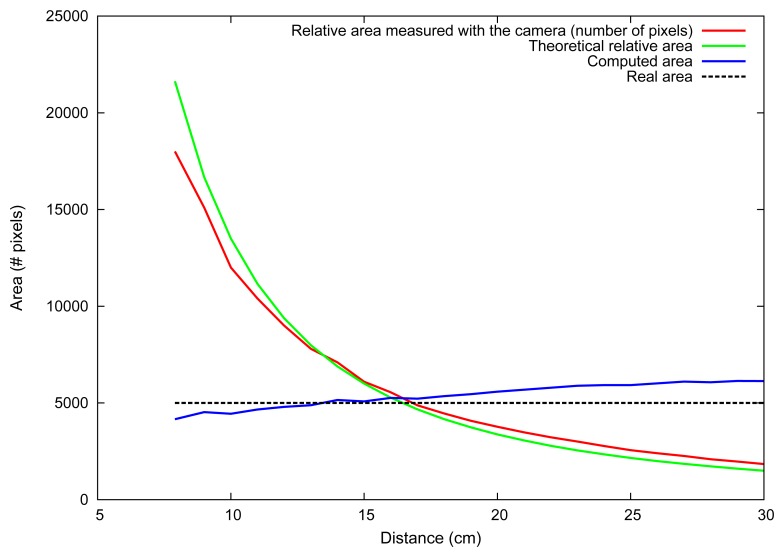
Area measurement (in pixels) of a circular object at different distances. Theoretical and experimental values are shown for the relative area (changes with distance) and the absolute area.

Equation (11) shown in Section 3 depicts the traditional pin-hole camera model, where *x* is the object height in the camera projective plane, *f* is the camera's focal length, *Z* is the distance from the camera to the object given by the distance sensor or laser pointer and *X* is the real size of the object. In Equation (12), we substitute *x* by the diameter of the object on the camera plane (2 · *r*) and *X* by the real height of the object 2 · *R* in the world. The minus sign means that the image formed in the camera plane is upside-down. Expanding Equation (12) with Equation (10), we find Equation (13), where *a_CI_* is the area of the circular object on the camera image obtained by the segmentation system and *A_CO_* is the area of the real object that we want to find. Simplifying Equation (13) yields Equation (14), which gives the object's area in pixels independently of the distance from the camera to the object. Figure 18 shows the theoretical size of an object of known size at different distances from the camera compared with real measurements made with our system. It also shows the computed area for this object at several distances, which is expected to be always the same. Note that the relative area measured with the camera (red line) follows the theoretical distances (green line) closely. In the same way, the black dotted line shows the real area, which is fixed, and the area measured with the camera is shown in the blue line, which ideally would be constant.

The area conversion from pixels to *cm*^2^ was approximated using ten circles of known size and fitting their values with a polynomial regression of order two, which is shown in Equation (15). To obtain the volume, Equation (10) is combined with the sphere volume equation shown in Equation (16).

(9) $$A_{C}=\pi \cdot r^{2}$$

(10) $$r=\sqrt{\frac{A_{C}}{\pi }}$$

(11) $$-\frac{x}{f}=\frac{X}{Z}$$

(12) $$-\frac{2\cdot r}{f}=\frac{2\cdot R}{Z}$$

(13) $$-\frac{2\cdot \left(\sqrt{\frac{a_{CI}}{\pi }}\right)}{f}=\frac{2\cdot \left(\sqrt{\frac{A_{CO}}{\pi }}\right)}{Z}$$

(14) $$A_{CO}\;(\text{pixels})=t=-\frac{a_{CI}\cdot Z^{2}}{f^{2}}$$

(15) $$A_{CO}\;(cm^{2})=-4.45\cdot 10^{-12}\cdot t^{3}+1.11\cdot 10^{-7}\cdot t^{2}+2.05\cdot 10^{-3}\cdot t-395\cdot 10^{-1}$$

(16) $$V_{S}=\frac{4}{3}\cdot \pi \cdot r^{3}=\frac{4}{3}\cdot \pi \cdot {\left(\sqrt{\frac{A_{CO}\;(cm^{2})}{\pi }}\right)}^{3}$$

**Figure 19.**
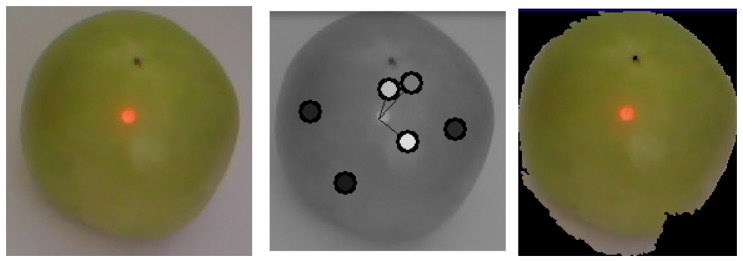
A green tomato image (left), with several laser seeds (middle) and resulting segmentation (right).

Figure 19 shows an image of a green tomato captured with the glove. The hand of the user pointed the laser to several parts of the tomato, which generated the seeds shown in the middle image of Figure 19. The resulting segmentation is shown on the right, and based on the pixel count, the area was measured with 90% accuracy and the volume with 74% accuracy. The segmentation time was 8 ms. It is important to note that these values are specific to this tomato, as the circle and sphere approximations might have different accuracies for other tomatoes. These results would not be adequate for other fruits; however, the proposed system, as a platform, is able to perform many other computer vision tasks to analyze other fruits using different computer vision algorithms. We remind that this is an example implementation of the possible uses of the platform, and the description and tests of other computer vision systems for fruit classification is outside the scope of this paper, which is focused on presenting the platform.

## 5. Conclusions

We have proposed a mobile sensing platform in the form of a wearable computer, a glove, for fruit classification. It is composed of several sensing devices, including cameras, pressure and temperature sensors, among others. A reference architecture is also presented, based on which several applications can be developed using subsets of the proposed system to address the needs of specific applications.

In order to make possible the implementation of this platform, we propose several novelties in this work, from which we highlight the use of the moving fovea approach with a laser to point, segment and compute geometrical properties of objects, and the usage of calibrated FSR sensors to sense pressure at fingertips.

As an application example, we also depict a new methodology that uses the glove for classifying fruits using data from several sensors. The experimentations have shown the feasibility of fruit grading using our system for several climacteric fruits.

The glove's price is about US$ 350.00 for the complete system, including cameras for the computer vision tasks. A sub-set of the platform was also built without cameras and computer vision capabilities. This simpler version costs only US$ 30.00 and includes sensor bending and pressure sensors. Thus, the battery life of this version is of more than ten work days. We think that this price would allow even small growers and harvesters to acquire such a glove to aid the execution of more uniform harvesting. Moreover, these prices are for a prototype—industrial production and scale would improve the system and decrease the price. Finally, although there is an increasing interest in robotic-based harvesting systems, many farmers, especially from developing countries, still cannot afford automated systems such as robots. Hence, our glove presents a possible solution in such cases, aiding harvesting with better objectivity and less variability.

We remark that the platform described in this article is suitable for a variety tasks. Of course, because it is a prototype and uses standard available electronics, enhancement can be done to improve the whole system performance. In this way, the presented glove can be considerably miniaturized using standard manufacturing techniques if products should be built using parts of the concepts presented here. Moreover, as computer and automation systems become more pervasive and ubiquitous, wearable systems such as gloves are a perfect assistant tool for hand-based tasks, since most of our daily tasks are done using hands.

## Figures and Tables

**Table 1.** Summary table of glove-based systems. Abbreviations: ACC = Accelerometers, FFS = Finger Flexion Sensors, Cam = Camera, IC = Internet Connectivity, HR = Heart rate sensor, TF = Tactile Feedback with vibration motor, PS = Pressure Sensor, SRR = Short Range Radio (Bluetooth, ZigBee, *etc.*), TS = Temperature Sensor.

**Category**	**Works**	**Main technologies used**
Medicine and rehabilitation	[15,30–33,37–40,52]	ACC FFS PS SRR Bio-signals IC HR TF TS
Input device for computers	[12–15,18–21,57]	ACC FFS SRR
Productivity, security and services	[17,48–50,54–56,58]	ACC RFID TS PS Cam
Assistive technology and health care	[22–25,27,40,41,43]	ACC Cam TS PS HR Speech synthesis TF
Behavior and daily life activities studies	[30,34–36,42,44–47]	ACCFFS RFID SRRGyro
Learning and training	[51–53]	TF PS

**Table 2.** Turgor pressures in Kilo-Pascal (kPa) for the measured fruits. The averages are computed based on 30 measurements.

**Fruit**	**Pressure (kPa)**	**Fruit 1**	**Fruit 2**	**Fruit 3**	**Fruit 4**
Tomato	Average	272	249	129	63
	Max/Min	275/271	255/241	133/126	64/63
	Standard deviation	1.7	3.5	1.7	0.5

Pear (Packans)	Average	269	179	156	85
	Max/Min	275/267	206/154	165/147	88/82
	Standard deviation	2.3	11.2	5.5	1.8

Banana (Prata)	Average	132	86	62	57
	Max/Min	138/121	94/76	62/61	59/52
	Standard deviation	4.4	4.4	0.3	1.4

Papaya	Average	257	186	73	54
	Max/Min	267/204	217/142	81/65	55/53
	Standard deviation	18.1	19.6	3.8	0.5

Guava	Average	267	222	161	81
	Max/Min	267/267	248/196	177/137	83/80
	Standard deviation	0	16.1	10.4	0.7

Mango (Tommy)	Average	259	158	85	70
	Max/Min	263/259	168/141	87/83	72/70
	Standard deviation	0.7	6.7	1.1	0.5

**Table 3.** Calibration data used to fit finger bending sensor values to spherical values.

**Raw 10-bit Sensor Reading (0–1,024)**	**Sphere diameter (mm)**
887	25
859	50
826	100
809	150

**Table 4.** Experimental results of computed diameter of spherical objects computed while the glove touches them. Results are based on 10-bit reading of ADC module and computed by a spline curve.

**Sensor Reading**	**Computed Diameter (mm)**	**Ground Truth (mm)**	**Error (%)**
872	37.98	35	2.98
860	49.03	50	0.97
862	47.13	50	2.87
870	39.77	40	0.23
865	44.32	45	0.68
844	66.73	65	1.73
842	69.51	70	0.49
825	102.51	100	2.51
812	146.49	150	3.51
